# Oxaliplatin-induced rhabdomyolysis in a postoperative rectal cancer patient: a case report and literature review

**DOI:** 10.3389/fphar.2026.1900733

**Published:** 2026-08-25

**Authors:** Lin Yang, Hongna Han, Zihua Guo, Ming Tian, Weiping Chang, Zhengrong Chen, Chao Sun

**Affiliations:** 1 Department of General Surgery, The First Affiliated Hospital of Xi’an Medical University, Xi’an, China; 2 Department of Neurology, The First Affiliated Hospital of Xi’an Medical University, Xi’an, China; 3 Department of Gastroenterology, The First Affiliated Hospital of Xi’an Medical University, Xi’an, China

**Keywords:** colorectal cancer, oxaliplatin, rhabdomyolysis, adverse drug reaction, chemotherapy-induced toxicity

## Abstract

This report describes the diagnosis and treatment of rhabdomyolysis associated with intravenous oxaliplatin administration in a patient undergoing postoperative chemotherapy for rectal cancer. The patient was a 73-year-old man who underwent laparoscopic robot-assisted radical resection for rectal cancer, pathologically staged as pT4aN1cM0 (IIIB). Postoperative chemotherapy was administered using the XELOX regimen. During the fourth cycle of chemotherapy, 2 h after completion of oxaliplatin infusion, the patient developed involuntary muscle twitching in both lower limbs accompanied by lower limb weakness. The family reported that the patient had previously experienced these symptoms following oxaliplatin infusion. This episode was accompanied by decreased urine output and dark, tea-colored urine. Laboratory tests revealed elevated creatine kinase (CK) levels of 2064.8 U/L. Following a multidisciplinary consultation, the patient was diagnosed with rhabdomyolysis and received symptomatic treatment, including hydration, alkalinization, and platelet-boosting therapy. After 5 days of treatment, creatine kinase (CK) levels returned to normal, and the patient was discharged. Following discharge, the patient discontinued chemotherapy. At the 8-month follow-up, all laboratory parameters remained within normal ranges.

## Introduction

Oxaliplatin is a third-generation platinum-based anticancer drug that induces cancer cell apoptosis by inhibiting DNA synthesis and replication, and is clinically used for the treatment of colorectal cancer, gastric cancer, and pancreatic cancer (O’Dowd et al., 2023). Colorectal cancer is one of the most common malignant tumors. Patients with locally advanced stage II/III rectal cancer may receive Total Neoadjuvant Therapy, TNT, which reduces the recurrence risk and improves organ preservation rates (National Comprehensive Cancer Network, 2026). Oxaliplatin is frequently combined with fluoropyrimidine agents for colorectal cancer chemotherapy (Gallois et al., 2024). Its dose-limiting toxicities mainly include peripheral neuropathy, gastrointestinal reactions, and hematologic toxicity (Ramanathan et al., 2003), while clinical reports of oxaliplatin-related rhabdomyolysis (RM) are rare. RM is a life-threatening complication. Striated muscle cell damage from various factors releases toxic cellular contents and myoglobin into the systemic circulation, which may cause acute kidney injury in severe cases (Nance and Mammen, 2015; Bosch et al., 2009). Early diagnosis and timely treatment are critical to improve the prognosis of patients with RM. This case report presents the diagnosis and treatment of RM in a rectal cancer patient following intravenous oxaliplatin infusion. It aims to improve clinicians’ recognition of this rare adverse reaction, summarize key clinical monitoring and management points, and provide practical references for the clinical management of similar cases.

## Case presentation

### Medical history summary

The patient is a 73-year-old male who underwent laparoscopic robot-assisted radical surgery for rectal cancer 3 months ago, with a pathological stage of pT4aN1cM0 IIIB. Postoperatively, he received regular chemotherapy with the XELOX regimen: oxaliplatin 240 mg IV infusion on day 1; capecitabine 1,500 mg orally, twice daily, 30 min after meals, from day 2 to day 15, with a 21-day cycle. He has a history of chronic atrophic gastritis and denies hypertension, diabetes, heart disease, cerebrovascular disease, as well as food or drug allergies. He has no history of smoking, alcohol consumption, or travel. The patient denied any recent falls, trauma, strenuous exercise, dehydration, or use of relevant medications. There is no record of related diseases in his family, and no history of infectious or hereditary diseases.

### Symptoms and signs

Upon admission, the patient’s vital signs were stable: T 36.2 °C, P 66 beats/min, R 18 breaths/min, BP 121/75 mmHg. After receiving the fourth cycle of chemotherapy, 2 h following the intravenous infusion of oxaliplatin injection, the patient experienced involuntary muscle twitching in both lower limbs, accompanied by weakness, and was unable to walk, with muscle strength of 5 in both upper limbs and 5 in both lower limbs. Pain sensation in all four limbs was intact. Urine output decreased, urine appeared dark tea-colored, and there was no percussion pain in the renal area. Blood tests showed elevated creatine kinase (CK) at 2064.8 U/L, creatine kinase-MB (CK-MB21.7 U/L, potassium (K) 3.23 mmol/L, calcium (Ca) 1.95 mmol/L, fibrinogen (Fbg) at 2.31 g/L, Phosphorus (P) 0.49 mmol/L, Whole blood lactate 4.08 mmol/L, Whole blood base excess −3.21 mmol/L, and creatinine (Cr) 0.87 mg/dL, with no myoglobinuria.

### Diagnostic method

A multidisciplinary consultation excluded common RM triggers: falls, trauma, exertion, dehydration and thrombotic muscle ischemia. No other myotoxic drugs were involved, as the patient took none recently. After excluding these factors, we reviewed her history, symptoms, lab results, bilateral lower limb Doppler ultrasound and symptom onset timeline. We confirmed oxaliplatin toxicity caused her RM (Nance and Mammen, 2015).

### Treatment method

**FIGURE 1:**
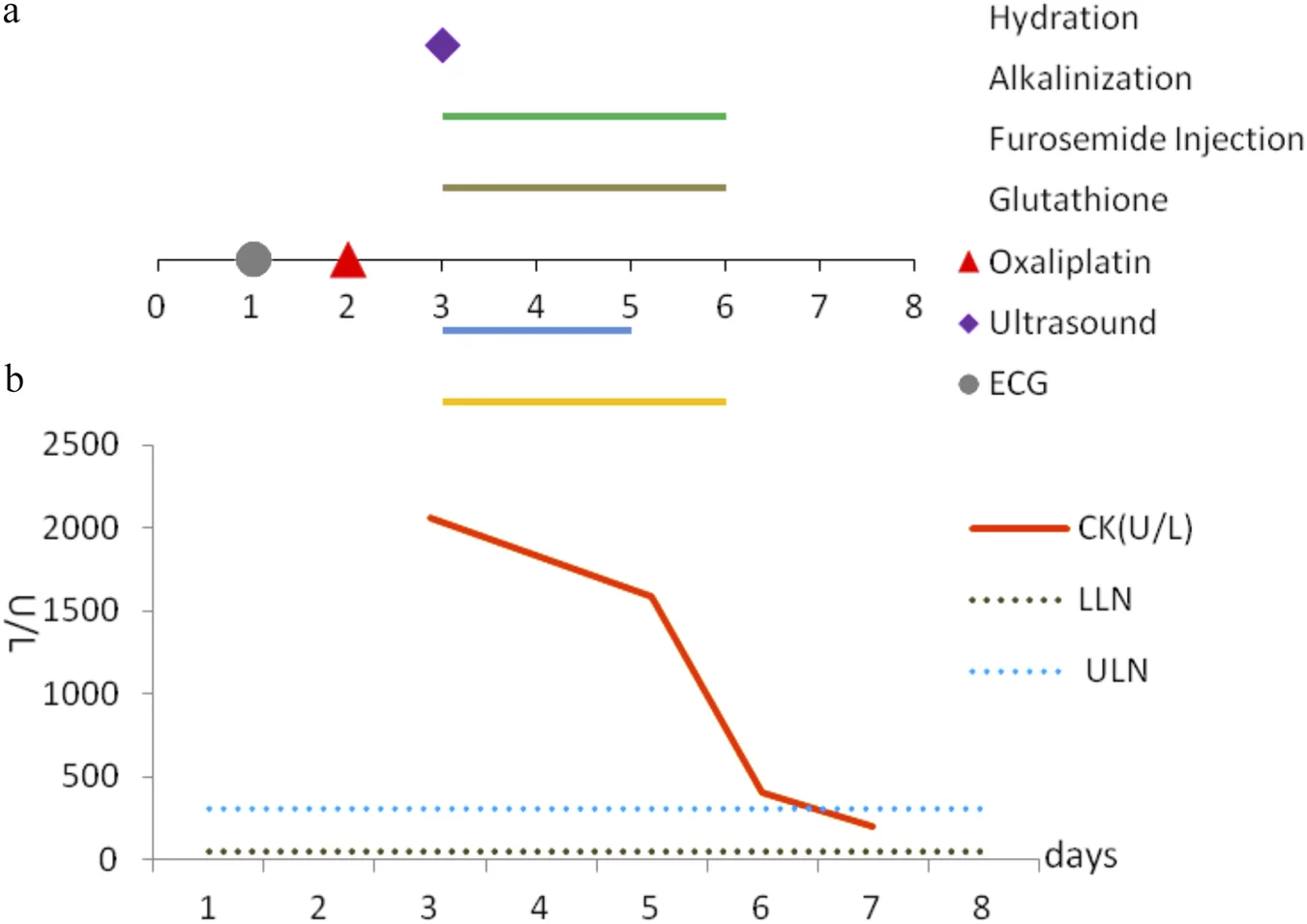
Timeline of interventions and CK level trends. **(a)** Timeline of clinical events and supportive interventions. Day 2 of hospitalization corresponds to the day of the fourth chemotherapy cycle. ECG on day 1 and ultrasound on day 3 were both normal. On day 3, the patient was diagnosed with rhabdomyolysis and received supportive treatments including hydration, alkalinization, furosemide, and glutathione. **(b)** Serial changes in serum CK levels during hospitalization. CK peaked at day 3 (2064.8 U/L) and gradually declined thereafter. The normal reference range was 50–310 U/L (LLN: lower limit of normal; ULN: upper limit of normal).

After multidisciplinary consultation, symptomatic treatment such as hydration, alkalization, and platelet elevation was provided. The detailed diagnosis and treatment process is shown in Figure 1.

### Clinical outcome

After 5 days of active treatment, the patient’s lower limb pain gradually eased, muscle strength gradually returned to normal, urine volume returned to normal, and the urine color became light yellow and clear. Creatine kinase levels returned to normal, and no acute kidney injury occurred. The patient was successfully discharged on the 8th day after admission. On follow-up outpatient visits 3 days, 1 month, 2 months, and 8 months after discharge, blood routine, liver and kidney function, electrolytes, and myocardial enzymes were all within the normal range.

## Discussion

Many drugs may cause RM in clinical practice. Common culprits include HMG-CoA reductase inhibitors, fibrate lipid-lowering agents, new-generation quinolone antibiotics, antipsychotics, antidiabetic agents, vasoprotective drugs, anti-Parkinson medications, anesthetics, muscle relaxants, nonsteroidal anti-inflammatory drugs (NSAIDs), and medications that cause electrolyte disorders such as hypokalemia. Of these, HMG-CoA reductase inhibitors and fibrates are the most frequently reported causative agents (Hohenegger, 2012).

Oxaliplatin-induced RM is rare in clinical settings, and no epidemiological incidence data are available to date. A literature search identified 11 published cases of oxaliplatin-associated RM. Combined with the present case, the total number reached 12, and most previously reported patients were of Asian ethnicity. The patients ranged in age from 30 to 73 years, with a male-to-female ratio of 5.5:1. The number of oxaliplatin cycles prior to symptom onset ranged from 1 to 16, with a median of 3. Most patients developed symptoms within the first several chemotherapy cycles, and a minority presented complaints only a few hours after drug administration. Primary malignancies included rectal cancer in 9 patients, colon cancer in 2 patients, and combined gastric cancer in only 1 patient. In terms of tumor staging, 7 patients had stage IV disease and 3 had stage III disease. Among the 12 total cases: 4 patients recovered 4–56 days after onset; 2 patients completed subsequent adjuvant chemotherapy without oxaliplatin; 2 patients died of acute renal failure on day 2 and day 51 after symptom onset, respectively; 1 patient died of massive hemorrhage from primary colorectal cancer on day 94; 1 patient died of intra-abdominal local recurrence of colorectal cancer on day 251 (Table 1). Most previously documented cases involved colorectal cancer, and more than half of these patients had advanced-stage tumors. Although oxaliplatin is widely applied for various gastrointestinal malignancies such as gastric and pancreatic cancer, rhabdomyolysis predominantly occurs in patients with colorectal cancer. Additionally, poor clinical outcomes are associated with peak creatine kinase elevation, renal failure, respiratory failure, laryngeal edema, or dysphagia.

**TABLE 1:** Cases of oxaliplatin-induced RMreported.

Case	Country	Age	Gender	Diagnosis	Stage	Primary tumor	Comorbidity	Time of occurrence of complications	Symptom	Serum CK level (U/L)	Serum creatinine (mg/dL)	Serum myoglobin level (ng/ML)	Laryngeal edema	Respiratory failure	Treatment	Treatment days	Follow-up
Case 1 (Pissarra et al., 2019)	Portugal	52	Male	Colon cancer	III	Rectosigmoid carcinoma	No	Cycle 2	Distal muscle pain and weakness, with a total inability to walk	8,264	2.35 mg/dL	NA	No	No	Hydration-n and alkali-nization	7	Adjuvant chemotherapy with capecitabine alone for six cycle-s more, with no toxicity
Case 2 (Katsumata et al., 2023)	Japan	50	Male	Gastric and ascending colon cancers	Not reported	Gastric cancer, ascending colon cancer	No	Cycle 11	Weakness	2031	Not reported	Not reported	No	No	Outpatient treatment without a specific regimen	Not reported	Adjuvant chemotherapy was completed without oxaliplatin Without recurrence for 9 months after the surgery
Case 3 (Maruoka et al., 2017)	Japan	73	Male	Transverse colon cancer, Sigmoid colon cancer, Metastatic liver cancer	IV	Transverse colon cancer, Sigmoid colon cancer	Hypertension	Cycle 1 Several hours afterdrug administration	Proximal limb pain and weakness	8,632	0.75 mg/dl	765.7	Yes	Yes	Hydration	112	Intraperitoneal metastases on day 251 after discontinuation of cancer therapy
Case 4 (Zhang et al., 2022)	China	55	Male	Rectal cancer	Not reported	Rectal cancer	No	Cycle 1	Lower limb pain and weakness	1,453	2.17	>1,000	NO	NO	Hydration-n and alkali-nization	4	Subsequent chemotherapy was not mentioned No adverse events during 3-week follow-up
Case 5	Japan	70	Male	Colon cancer	IV	Colon cance	Diabetes mellitus	Cycle 3	Not reported	15,658	Not reported	15,000	Yes	Yes	Not reported	Not reported	Death
Case 6	Japan	60	Male	Rectal cancer	IV	Rectal cancer	None	Cycle 16	Not reported	4,805	Not reported	Not reported	NO	NO	Not reported	Not reported	Death
Case 7	Japan	50	Male	Rectal cancer	IV	Rectal cancer	None	Cycle 4	Not reported	2,992	Not reported	Not reported	Yes	Yes	Not reported	Not reported	Death
Case 8	Japan	70	Male	Rectal cancer	IIIb	Rectal cancer	Hypertension	Cycle 1	Not reported	2,423	Not reported	280	NO	NO	Not reported	Not reported	Alive
Case 9	Japan	30	Female	Colon cancer	IV	Colon cancer	Myotonic dystrophy	Cycle 3	Not reported	2,314	Not reported	220	NO	NO	Not reported	Not reported	Alive
Case 10	Japan	60	Male	Rectal cancer	III	Rectal cancer	Hypertension	Cycle 1	Not reported	1,679	Not reported	Not reported	NO	NO	Not reported	Not reported	Alive
Case 11	Japan	50	Female	Rectal cancer	IV	Rectal cancer	None	Cycle 4	Not reported	Not reported	Not reported	Not reported	NO	NO	Not reported	Not reported	Alive
Case 12*	China	73	Male	Rectal cancer	IV	Rectal cancer	Chronic atrophic gastritis	Cycle 4 Onset at 2 h post-administration	Involuntary lower limb muscle twitching with weakness	2064.8	0.87	Not tested	NO	NO	Hydration-n and alkali-nization	5	Chemotherapy discontinued; no recurrence at 8-month follow-up

* Present case.

Cases 5–11 were sourced from Yakult Honsha Co., Ltd. (the marketing authorization holder of oxaliplatin in Japan), comprising seven reported cases of oxaliplatin-induced RM, in Japan.

CK, creatine kinase; Cr, creatinine; NA, not available.

**FIGURE 2:**
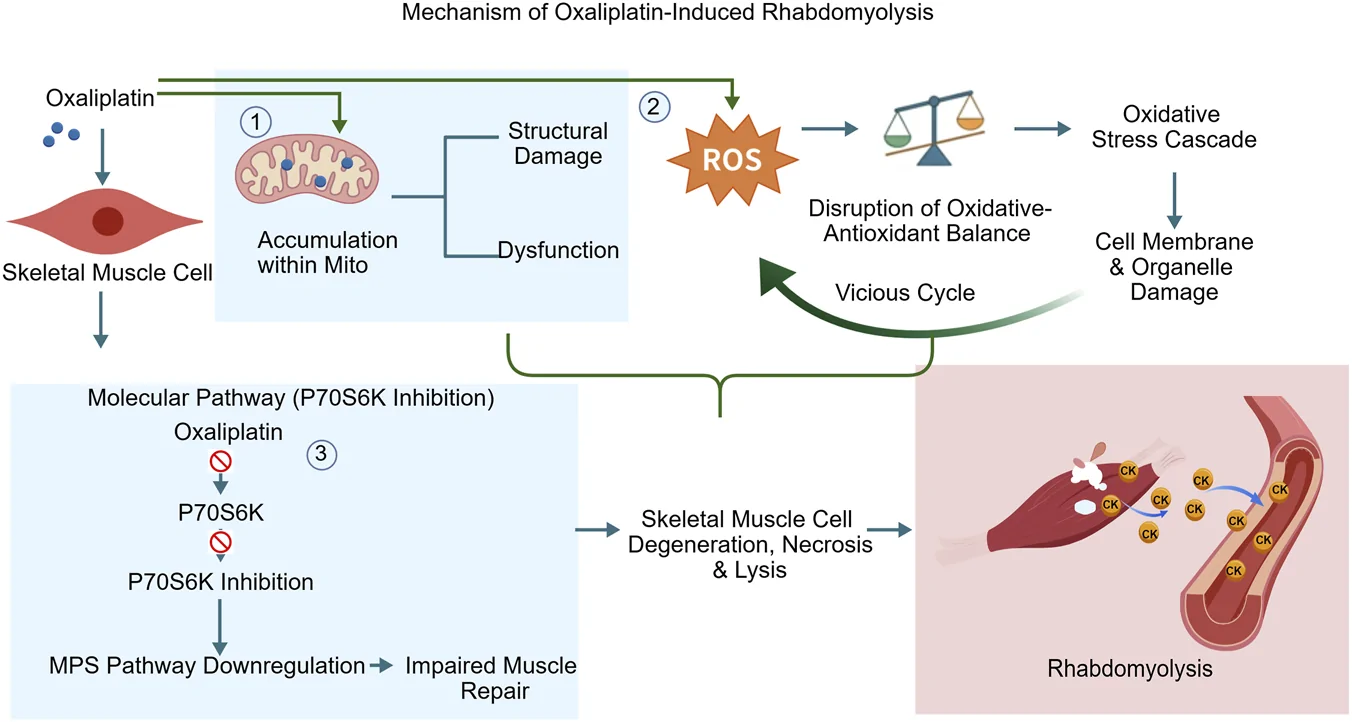
Mechanism of Oxaliplatin-Induced Rhabdomyolysis. Schematic diagram illustrating the pathway of muscle cell injury triggered by oxaliplatin. Schematic created using BioGDP (https://biogdp.com) with official academic publication license.

The pathogeneses of drug-induced RM (Hohenegger, 2012; Tang et al., 2025) include direct myotoxicity induced by drug overdose, immune-mediated inflammatory myopathy, indirect skeletal muscle injury, and allergic reactions. This case describes oxaliplatin-induced RM. The underlying mechanism is as follows: after intravenous infusion, oxaliplatin enters the bloodstream. Circulating free oxaliplatin crosses vascular endothelial cells and is transported into skeletal muscle cells via OCT2/OCT3 transporters on the sarcolemma (Yonezawa et al., 2006). Platinum ions penetrate the sarcolemma and accumulate within mitochondria. On one hand, they disrupt mitochondrial structure and function, thus inducing oxidative stress injury. On the other hand, they disrupt calcium homeostasis, activate hydrolytic pathways, and damage myofibril architecture. Furthermore, oxaliplatin inhibits p70S6K and rpS6 to decrease myoprotein synthesis and weaken the self-repair capacity of muscle cells. In the present case, the patient completed 4 cycles of chemotherapy. Cumulative oxaliplatin toxicity gradually exacerbated muscle damage and eventually triggered RM (Cheregi et al., 2015; Sorensen et al., 2016; Sorensen et al., 2017) (Figure 2).

In addition, previous studies have found that prolonged lithotomy position, Trendelenburg position, and CO_2_ pneumoperitoneum during colorectal surgery can lead to lower limb venous congestion and progressive elevation of intracompartmental pressure within osteofascial compartments. These changes result in hypoperfusion of skeletal muscle, trigger muscle injury, and raise CK levels. However, this injury alone is insufficient to trigger RM (T suchiya et al., 2020; Kusunoki et al., 2024). The patient in this case underwent robotic-assisted laparoscopic radical resection for rectal cancer. RM developed after completion of the fourth cycle of chemotherapy. Myotoxicity from oxaliplatin played a dominant role. Intraoperative muscle injury only acted as a predisposing factor. These two factors work together to synergistically worsen muscle cell injury and lysis.

RM is a severe syndrome associated with muscle injury, typically caused by traumatic or non-traumatic damage to skeletal muscle. Its pathophysiological basis involves the destruction of muscle membranes and the release of intracellular contents into the bloodstream (Sauret et al., 2002). While the mortality rate for RM is relatively low, approximately 10%–40% of cases may develop acute kidney injury if not treated promptly, with a mortality rate as high as 80% (Chatzizisis et al., 2008). Only 10% of patients present with typical symptoms, including muscle swelling, pain/cramps, weakness, and darkening of urine color; common sites of involvement include the calves, proximal thighs, lower back, and large muscle groups (Stahl et al., 2020; Cervellin et al., 2017). The diagnosis of RM requires a detailed personal medical history, clinical symptoms, and laboratory tests. Among these, serum CK levels are a sensitive indicator of skeletal muscle damage, while plasma and urine myoglobin measurements are generally used in the early stages of the disease (Huerta-Alardín et al., 2005). Ostrowski et al. (2023) introduced novel monitoring methods for drug-induced muscle injury and rhabdomyolysis—a panel of muscle injury biomarkers. This includes new immunoassay-based marker panels and microRNAs/muscle-specific microRNAs, such as skeletal muscle troponin I, myosin light chain 3, fatty acid-binding protein 3, CK measured by mass spectrometry, and miR-206. Currently, most studies use CK cut-off values to classify RM into mild, moderate, and severe categories, with cut-off values of 1,000 U/L, 5,000 U/L, and 15,000 U/L^6^. Elevated CK levels or failure of CK to decrease indicate ongoing muscle injury or the development of renal failure, with a poor individual prognosis (Zimmerman and Shen, 2013).

For the management of drug-induced RM, early fluid resuscitation serves as the cornerstone of treatment. Guidelines recommend 0.9% normal saline, and early fluid supplementation is correlated with favorable clinical outcomes (Ron et al., 1984). Urine output should be closely monitored during fluid therapy, with a target of 200–300 mL per hour. Urine alkalization is proposed based on animal model data. Isotonic bicarbonate solution is the first-line intravenous resuscitation fluid for patients with severe renal injury-related metabolic acidosis, and serum electrolytes should be continuously monitored throughout administration. As an osmotic diuretic, mannitol is mainly indicated for RM induced by trauma and crush injury (Grover and Sripathi, 2026). In the present case, 0.9% normal saline was given for fluid resuscitation before serum CK levels peaked, and furosemide injection was used for diuresis. These measures improved renal perfusion and averted acute kidney injury. Once the diagnosis was confirmed, all chemotherapy was halted immediately to prevent progressive muscle injury, and mecobalamin was administered for peripheral neuroprotection. Laboratory examinations showed metabolic acidosis and liver dysfunction in this patient. Prior studies have verified that sodium bicarbonate can correct mild-to-moderate acidosis (Chua et al., 2011), and antioxidants lower the risk of acute kidney injury in RM patients (Bosch et al., 2009). Accordingly, sodium bicarbonate injection was used to reverse metabolic acidosis under close monitoring of blood acid-base parameters and electrolytes. Since the patient and their family refused blood purification, glutathione was provided for liver protection to limit the progression of muscle and visceral injury amid drastically elevated muscle enzymes.

Traumatic RM secondary to trauma or crush injury is prevalent clinically (Giles et al., 2024), while oxaliplatin-induced drug-associated RM remains uncommon. We compared the present case with traumatic RM in clinical features, metabolic parameters and treatment strategies, aiming to facilitate early recognition, definite diagnosis and prompt treatment by clinicians.

### Clinical features

The patient merely suffered from bilateral lower limb cramping and asthenia with mild local complaints, showing a striking distinction from traumatic RM, which presents prominent limb swelling and severe pain at early onset.

### Metabolic parameters

Early laboratory changes of traumatic RM include hyperkalemia, hyperphosphatemia, metabolic acidosis, sharply elevated CK (>5,000 U/L) and myoglobinuria. Conversely, this patient had hypokalemia (3.23 mmol/L), hypophosphatemia (0.49 mmol/L) and gradually increasing CK (peak 2064.8 U/L). No myoglobinuria was detected during the whole course, though metabolic acidosis (lactate 4.08 mmol/L) existed.

### Treatment strategies

In addition to general supportive care, traumatic RM demands urgent decompression of compressed skeletal muscles and immediate correction of hyperkalemia. For our patient, oxaliplatin was withdrawn instantly, and XELOX chemotherapy was terminated. Serial monitoring of CK, hepatic and renal function was performed during regular follow-up, with sustained surveillance for myotoxic manifestations.

In conclusion, this case report describes one patient with oxaliplatin-induced RM. It aims to alert clinicians to watch out for mild local somatic symptoms and summarize previously published cases. Although oxaliplatin is widely used in multiple gastrointestinal malignancies such as gastric and pancreatic cancer, RM predominantly develops in patients with colorectal cancer. Clinicians should remain highly vigilant to achieve early diagnosis and deliver timely standardizsectionbed treatment.
